# Quality, Stability, and Safety Data of Packed Red Cells and Plasma Processed by Gravity Separation Using a New Fully Integrated Hollow-Fibre Filter Device

**DOI:** 10.1155/2009/175234

**Published:** 2010-02-04

**Authors:** T. Brune, K. Hannemann-Pohl, K. Nißle, N. Ecker, H. Garritsen

**Affiliations:** ^1^University Children's Hospital, Gerhart-Hauptmann- Strasse 35, 39108 Magdeburg, Germany; ^2^Department of Transfusion Medicine, Marienkrankenhaus, Hamburg, Germany; ^3^Institute for Clinical Transfusion Medicine, Städtisches Klinikum, Braunschweig gGmhG, Germany

## Abstract

*Background*. We developed a completely closed system based on gravity separation without centrifugation steps for separation of whole blood. With this new system we compared quality and stability of the processed blood components (PRC and plasma) with respect to classical preparation. Furthermore the cost-effectiveness of this hollow fibre system was evaluated. *Study Design and Methods*. Whole blood collections of 15 regular blood donors were used for component preparation using the U shaped hollow fibre filter device. Results were compared to 15 whole blood preparations using centrifugation. The following parameters were evaluated: total hemoglobin, leukocyte counts, the serum concentration of total protein, lactate dehydrogenase (LDH) and potassium. Furthermore ATIII, vWF and F VIII were analyzed at different timepoints. *Results*. packed red cells: the data directly after separation and after 42 days of storage are in line with the guidelines of the council of Europe. *Plasma*. all plasma quality data are in line with the guidelines of the council of Europe for quality assurance of plasma, except for a low protein amount (factor 0.75). *Conclusion*. Separation of whole blood on a clinical scale in this new closed system is feasible, however the plasma protein content must be optimized.

## 1. Introduction

Separation of whole blood into the different blood components is a standard procedure in transfusion medicine. This separation is either performed by centrifugation using programmable centrifuges and automated blood-component preparation systems after collecting whole blood or during the collection of blood in the case of cellular apheresis. In both cases an extensive infrastructure (central facility, expensive equipment, and well-trained staff) is required for the successful processing of the blood. Besides the necessary equipment, labor-intensive manual processes are necessary in the case of whole blood separation or careful supervision of the apheresis process in the case of cellular apheresis. We investigated if the above-mentioned separation methodologies could be replaced by a closed-system gravity filtration.

There were several attempts to replace the centrifugation and additional separation method using simple gravity filtration. The first study on the separation of whole blood to PRCs and plasma using a hollow-fibre system was performed by Sekiguchi et al. [1]. By changing the filter systems, integrating leukocyte filters and using a storage medium for extended storage, different authors were able to show that it is possible to process PRCs and plasma using gravity separation with comparable quality and stability data to those processed by classic centrifugation/apheresis methods. Nevertheless, the problem with these first attempts was the low concentration ability of the filter system with a resulting low HCT in the PRCs and a reduced plasma recovery. This low concentration ability mainly results from the relatively high flow rate through the linear filter system [2–5] preventing an effective separation into plasma and cellular components. 

**Figure 1:**
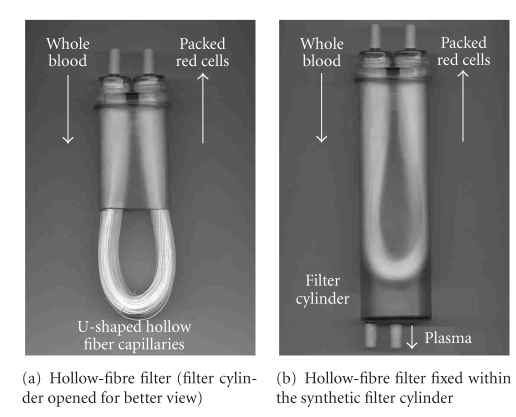
Figures 1(a) and 1(b) show the hollow-fibre filter in more detail. Each hollow-fibre filter is made of 600 capillary membranes, produced from polyethersulfon with a thickness of 100 *μ*m ± 25 *μ*m. The capillaries are U-shaped (Figure 1(a)) fixed within the synthetic filter cylinder (Figure 1(b)) and moistened with a sodium chloride solution. The capillaries have a pore size of 0.5 *μ*m ± 0.1 *μ*m. The total length of the capillary amounts to 175.5 m with a diameter of 300 *μ*m ± 40 *μ*m. The hydrostatic pressure of the donation bag fixed above the filter is enough to press the erythrocytes into the erythrocytes bag. During the separation process the plasma penetrates the capillary membranes, is sampled in the filter cylinder and flows down to the plasma bag. Because of the cells' volume the erythrocytes are not able to penetrate the membrane and flow along the U-shaped capillary to the upper exit in the erythrocytes bag hung at the same height and prefilled with the 100 mL PAGGS-M additive solution.

To improve the separation potential a new hollow-fibre system was designed with hollow fibres enclosed in a plastic casting in the shape of a stirrup (U shape), which was separated, by polyurethane, into compartments for plasma (outside the capillaries) and red cells (within the fibres). Details of the hollow-fibre filter are given in Figure 1. This preliminary filter design was incorporated into a blood separation device and was tested by Heim [6], Stienstra et al. [7], and Hornsey et al. [8]. They concluded that whole blood separation using the hollow-fibre technique is simple and straightforward to use; however, minor changes are required to make the procedure easier to incorporate into routine use. 

Based upon the practical experience of Hornsey we modified the original hollow-fibre device in an attempt to improve its performance.

In the preliminary system evaluated by Hornsey et al. the filter system and the donation bag were separate and the hollow fibre system was dry. A Pall leukocyte filter was used for leukocyte depletion. Before separation the hollow-fibre filter had to be moistened carefully with sodium chloride and the donation bag had to be sealed sterile to the blood donation bag. Based on the experience with this preliminary system we developed a complete integral pack closed system containing anticoagulant and an additive solution attached directly to the device and with filters premoistened with sodium chloride. In addition, we replaced the Pall leukocyte depletion filter by a Fresenius leukocyte depletion filter. In the present study we present the quality and stability data of PRCs and plasma which were processed with this new clinical system. To evaluate the possible cost-effectiveness, we compared both systems with respect to hands-on time.

## 2. Donors, Material, and Methods

### 2.1. Donors

15 donors were stochastically selected from regular blood donors of the Department of Transfusion Medicine, Marienkrankenhaus, Hamburg, Germany. All donors were informed about the clinical trial and the use of the donated blood. All participating donors gave their informed consent.

### 2.2. Blood Collection

All blood donations were performed according to the standard guidelines for blood donations in Germany. In each donor the following parameters were measured: total hemoglobin (Hb), leukocyte count, the serum concentrations of total protein, lactate dehydrogenase (LDH), and potassium. Furthermore, the activities of the coagulation factors antihrombin III (AT III), von Willebrand factor (vWF), and factor VIII (F VIII) were analyzed. The analyzers we used for these investigations are presented in Table 1. All measurements were performed according to the recommendations of the manufacturers of the analyzers used. 

After careful desinfection and successful punction of the cubital vein we collected 450 mL blood from each donor using the integrated hollow-fibre system. During the blood collection the donation bag was placed on a standard blood-mixing device (NPBI Compomixer M2). Immediately after the blood collection the inlet tubing of the donation bag was sterile sealed.

### 2.3. Blood Separation

**Figure 2:**
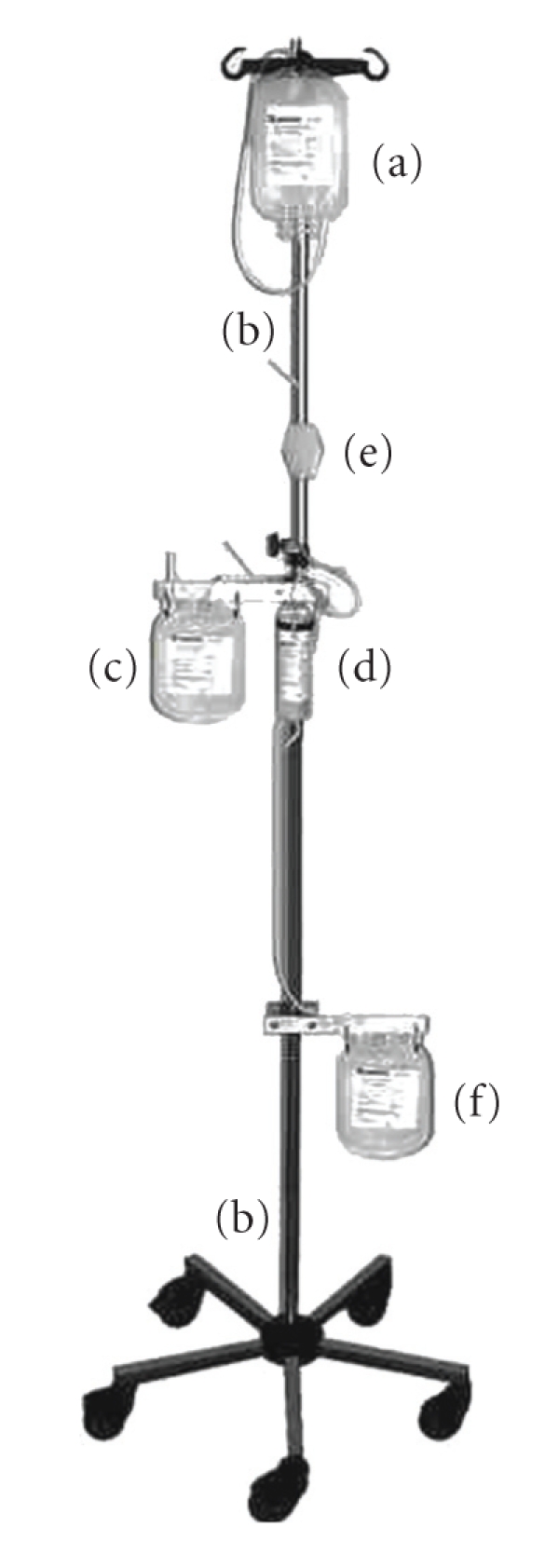
Complete gravity-separation system. (a) Donation bag prefilled with 63 mL CPD, (b) Mean stand, (c) Erythrocyte bag prefilled with 100 mL extended storage medium (PAGGS-M), (d) Hollow-fibre filter, (e) Leukocyte filter, (f) Plasma bag.

Before blood separation the donated whole blood unit was left to rest at ambient temperature (about 20°C) for 2 hours in order to allow phagocytosis for free bacteria. The details of the closed hollow-fibre separation system are given in Figure 2. The gravity-separation procedure is performed as follows. First the donation bag containing 63 mL CPD (1000 mL of CPD contains 3.27 g of citric acid·H_2_O, 26.3 g of sodium citrate·2H_2_O, 25.5 g of glucose·H_2_O, and 2.51 g of NaH_2_PO_4_·H_2_O) is hung on the upper hook of the main stand (b). Then the packed red cells (PRCs) bag (c) prefilled with extended storage medium (100 mL PAGGS-M) (1000 mL of PAGGS-M contains 9.400 g of glucose·H_2_O, 1.255 g of NaH_2_PO_4_·H_2_O, 1.432 g of NaHPO_4_·H_2_O, 0.194 g of adenine, 0.408 g of guanosine, 10.000 g of mannitol and 4.210 g of sodium chloride) and the separation filter (d) is hung on the middle attachments both exactly on the same level. After all clamps and inline break valves have been opened the blood flows from the donation bag through the leukocyte filter (e) and subsequently into the hollow-fibre system. While the leukocyte-depleted blood is slowly running through the inner space of the U-shaped hollow fibres the plasma is diffusing through the porous walls with the result that the blood is separated into plasma and erythrocytes. The blood flow can be regulated via a roller clamp, and the flow rate was regulated via a drip chamber. The cell-free plasma flows down into the plasma bag (f) and the erythrocytes to the erythrocytes bag. After approximately 40 minutes the separation is finished. After completion of the separation the PRC bag and the plasma bag were sealed and disconnected from the separation set. All plasma preparations were frozen immediately and stored at −30°C (Blood Plasma Freezer: UF 40-300S, Colora GmbH, Lorch, Germany). For quality control testing of the PRCs we performed an automatic blood cell count and the free hemoglobin concentration was measured. The hemolysis rate was calculated according to the formula (100 – Hct) × free HB/Hb. After processing the 15 PRCs were stored for 42 days at +4°C in a regular blood bank refrigerator (Blood Bank Refrigerator BL-720, Philipp Kirsch GmbH, Offenburg, Germany) After 42 days of storage all quality controls described above were repeated in the PRCs and sterility control was performed. For quality control testing of the plasma, concentrations of total protein, LDH, potassium and fibrinogen and also the residual leukocytes count were performed. In addition, the activity of the coagulation factors AT III, vWF, and F VIII was determined. The plasma was frozen at −30°C according to a standard protocol. After 366 days of storage the frozen plasma was thawed and F VIII activity was measured again.

### 2.4. Cost-Effectiveness

To compare the presented method of blood separation by gravity with the classic method using whole blood and centrifugation as to its cost-effectiveness, we analyzed the hands-on time of each work step in both procedures (Centrifugation/Separation *n* = 6; Separation by gravity *n* = 3). Only the real blood processing time was included in the evaluation; the blood sampling time is the same for both methods and was therefore not considered. 

## 3. Results

### 3.1. Donor Parameters

The results of the donors' blood parameters are given in Table 2.

### 3.2. Packed Red Cells

The quality data of the processed PRCs directly after separation are given in Table 3(a). After 42 days of storage the content of free Hb was 1.2 g/L (range: 0.8 g/L ± 1.7 g/L), which corresponds to a mean lysis rate of 0.7% (range: 0.5%–0.9%) (Table 3(b)). All values are in line with the guidelines of the Council of Europe [9] for the quality of PRCs (Table 4). The HCT of the PRC directly after separation can be calculated from the mean total volume (346 mL) and the mean HCT (0.54) of the PRCs and the knowledge of the added SAG-M amount according the formula 346 mL × 0.54/(346 mL–100 mL) = 0.76. The donor's original HCT of 0.43 is diluted by 63ml CPDA in the primary bag to an HCT of 0.37(450 × 0.43/(450 + 63)). Based on these values the calculated concentration ability of the filter is 76/37 = 2.05.

### 3.3. Plasma

The quality data of the plasma preparations are given in Table 5(a). The protein content is reduced by a factor of about 0.75 compared to the values of the initial donor blood (Table 1). The plasma activity of antihrombin III, von Willebrand factor, and factor VIII correlates to the protein content reduction and was approximately 0.8. The residual leukocyte count in the plasma was at the lower detection limit (below 2000/unit) and the plasma contained no measurable free hemoglobin. The factor VIII activity after one year of storage was 0.57 with a range of 0.35–0.97 (Table 5(b)). This corresponds to 0.78 of the activity before storage (range: 0.69–0.88). Serum electrophoresis of randomly selected plasma shows a normal protein fraction distribution within the reference values (Table 6). All plasma quality data are in line with the guidelines of the Council of Europe for the quality assurance of plasma (Table 7), except for the low protein amount.

### 3.4. Cost-Effectiveness

The analysis and comparison of the different steps of both ways of whole blood processing are given in Table 8 with the time necessary for a certain step given in minutes. This table shows that the processing of whole blood into PRCs and plasma takes a total of 70 minutes with the centrifugation/separation method and 57 minutes with the gravity-separation method. Both the centrifugation process and the gravity-separation process themselves are independent of a technologist and are times in which the technologists can perform other tasks. We therefore postulate that a technologist is needed for 50 minutes using the classic method and only 12 minutes using the gravity-separation method. 

## 4. Discussion

Our study demonstrates that whole blood can be separated efficiently into its components using a gravity-driven completely closed hollow-fibre system. The quality and the stability of the PRCs and plasma thus processed are comparable to the quality and the stability of blood components processed in the classic way and correspond to the German and European guidelines for the preparation, use and quality assurance of blood components. However, the protein concentration of the processed plasma is still too low. The reasons for this protein loss are not completely understood. We speculate on an interaction of the plasma protein with the capillary membranes of the hollow fibre (polyethersulphone) causing absorption of plasma protein. Taking into account that the total free length of the hollow fibre is 175.5 m, minimal absorption could lead to a measurable difference in plasma protein. A second mechanism could be by activation of the coagulation system (platelet activation?) due to contact with the artificial surface of the hollow fibre. Again this activation of the coagulation system might be minimal, but the total surface of the hollow fibre system could make the difference. We plan to work on these aspects by chemically modifying the surface of the hollow fibres to inhibit adsorption or activation. The hollow-fibre filter blood-separation device eliminates the need for either centrifugation or automated separation steps during the processing of whole blood into red cells and plasma components. A source of electrical power is not required and the closed system can be used anywhere, irrespective of environmental conditions. This means that an integral system for safe blood collection and processing is also within reach of under-resourced countries. However, other applications are focussed upon as well. Blood collections obtained at remote blood drives can be processed during the actual blood drive locally without the need for a speedy return to a central facility. The handling of the system is easy and handy in practice. The net working time for processing the blood components is lower compared to the centrifugation method, only requiring a small number of staff and no large, specialized machines. Only a heat melting clamp or clamping tongs for closing the tubes, a tubing roller and the usual cooling and freezing equipment are necessary for the production of the preparations from donor blood described above. 

The system has been in clinical use in different countries (Turkey, South Africa, and Mozambique, among others) for 2 years. To the authors' best knowledge no adverse effects related to the separation system have been observed. 

## Figures and Tables

**Table 1:** 

Parameter	Analyzer
Free Hemoglobin:	Analyzer Low Hemoglobin, Hemocue
Hematology:	Cell-Dyn 4000 and CellDyn 350, Abbott, USA
Total-Protein, Serum LDH, Serum potassium:	AU 640/AU 2700, Olympus
Von-Willebrand-Antigen:	Mini-VIDAS, Biomerieux, Paris, France
Fibrinogen, F VIII:	BCT, Dade Behring, Marburg, Germany
Residual leucocytes:	Epics XL, Coulter, USA

**Table 2:** Donors, initial values.

*N* = 15	Hb (g/dL)	Leukocytes (10^3^/*μ*L)	Total Protein (g/L)	LDH (U/L)	K^+^ (mmol/L)	AT III (% o. N.)	vWF-Antigen (% o. N.)	F VIII (% o. N.)
Mean ± SD	13.8 ± 1.1	5.2 ± 1.5	70 ± 4	176 ± 42	4.1 ± 0.2	104 ± 11	86 ± 24	98 ± 28
Range	11.2–15.2	3.5–8.5	62–77	122–290	3.7–4.7	93–119	58–118	57–149

**Table:** (a) Packed red cells directly after preparation.

*N* = 15	Volume (mL)	Hb Concen-tration (g/dL)	Hb/Unit (g)	Hct (%)	Residual Leukocytes/Unit (×10^6^)	Residual Protein/Unit (g)	K^+^ in Supernatant (mmol/L)	K^+^/Unit (mmol)	Free Hb (g/L)	LDH in Supernatant (U/L)
Mean ± SD	346 ± 19	17.4 ± 1.2	60 ± 6	54 ± 4	0.03 ± 0.00	3.3 ± 0.8	1.8 ± 0.2	0.28 ± 0.04	<0.03	346 ± 19
Range	326–402	14.3–19.0	47–73	43–59	0.03–0.04	2.0–4.9	1.4–2.0	0.21–0.32	<0.03	25–73

**Table:** (b) Packed red cells after 42 days of storage

*N* = 10	Free Hb (g/L)	Lysis rate
Mean ± SD	1.21 ± 0,33	0.69 ± 0.16
Range	0.8–1.7	0.5–0.9

**Table 4:** Requirements for PRC.

Parameters	Requirement	Observations
Haemoglobin im PRC	≥40 g/Unit	47–75 g/Unit
Residual Leukocytes in PRC	<1 × 10^6^/Unit	<0.05 × 10^6^/Unit
	No Leakage	No Leakage
Visual Examination	No Agglutination	No Agglutination
	No Change of Color	No Change of Color

**Table:** (a) Plasma directly after preparation.

*N* = 15	Volume (mL)	Total Protein Concentration (g/L)	Total Protein/Unit (g)	Residual Leukocytes/Unit (×10^6^)	Fibrinogen (mg/dL)	Fibrinogen/ Unit (mg)	AT III (% o. N.)	vWF-Antigen (% o. N.)	F VIII (% o. N.)
Mean ± SD	234 ± 12	51 ± 4	12.0 ± 1.2	0.02 ± 0.00	191 ± 41	450 ± 109	82 ± 9	82 ± 27	82 ± 27
Range	214–253	44–58	9.7–14.6	0.02–0.03	138–264	304–663	67–95	52–119	46–115

**Table:** (b) Plasma F VIII after 366 days of storage.

*N* = 15	F VIII (% o. N.)	% of activity before storage
Mean ± SD	57 ± 21	78 ± 5
Range	35–97	69–88

**Table 6:** Plasma electrophoresis directly after preparation.

	Results	Reference values
Total protein	58 g/L	60–80 g/L

Albumin	57%	55–67%
Alpha 1 Globulin	2.7%	2.1 –3.7%
Alpha 2 Globulin	9.4%	8.0–14.0%
Beta Globulins	10.8%	8.0–13.0%
Gamma	20.1%	9.0–20.0%

**Table 7:** Requirements for plasma preparations.

Parameters	Requirement	Observations
Volume fluctuation of plasma preparations	−10% ⋯ +10%	−7.8% ⋯ +9.0%
Residual leukocytes in plasma	<1 × 10^6^/Unit	<0.04 × 10^6^/Unit
	No leakage	No leakage
Visual examination	No agglutination	No agglutination
	No change of color	Clear, unclouded, amber coloured plasma

**Table 8:** Comparison of the mean processing times of the classical separation method and gravity separation.

Process activity	Centrifugation	Gravity Separation
Transport of the blood bags to the lab	2 min	2 min
Preparation of the leucocytes depletion	4 min	
Leukocytes depletion	15 min	
Preparation of the centrifugation step (cooling)	5 min	
Centrifugation	20 min	
Preparation of the blood separator	4 min	
Separation of the whole blood to PRC and Plasma	12 min	
Documentation	3 min	
Preparation of the PRC and Plasma for storage	5 min	
Preparation of the gravity Separation filter system		4 min
Gravity separation		45 min
Documentation		3 min
Preparation of the PRC and Plasma for storage		5 min
Sum	70 min	57 min
Minus centrifugation, respectively. gravity separation time (technician is free to perform other things)	20 min	45 min
Time technician is required for the processing	50 min	12 min
